# Examining the geographic distribution of six chronic disease risk factors for severe COVID-19: Veteran–nonveteran differences

**DOI:** 10.1177/17423953211028280

**Published:** 2021-09-05

**Authors:** Michelle McDaniel, Justin T McDaniel

**Affiliations:** 1 College of Adult and Graduate Studies, Colorado Christian University, Lakewood, USA; 2 School of Human Sciences, Southern Illinois University, Carbondale, USA; 3 Center for Alzheimer’s Research and Treatment, Southern Illinois University School of Medicine, Springfield, USA

**Keywords:** COVID-19, veterans, risk factors

## Abstract

**Objectives:**

We aimed to better understand where the prevalence of risk factors for severe COVID-19 occur, especially among veterans and nonveterans – populations that are given the opportunity to seek healthcare from separate entities.

**Methods:**

In this cross-sectional study, we use data from the SMART Behavioral Risk Factor Surveillance System to estimate the prevalence (i.e., survey-weighted %) of six risk factors for severe COVID-19 (i.e., chronic obstructive pulmonary disorder [COPD], asthma, diabetes, obesity, cardiovascular disease, and kidney disease) for veterans and nonveterans at the national level, in 155 metropolitan/micropolitan statistical areas, and in Veteran Integrated Service Networks (veterans only). We examine differences in these outcomes among veterans and between geographic areas using chi-square analysis or multivariable logistic regression.

**Results:**

In the national aggregate, veterans exhibited higher prevalence rates of COPD, diabetes, cardiovascular disease, and kidney disease than nonveterans, but not asthma and obesity. However, we show significant variation in the prevalence of risk factors for severe COVID-19 among veterans by geographic location.

**Discussion:**

This study provides a dataset that can be used by healthcare providers in order to prioritize prevention programming for veterans who may be at higher risk for COVID-19 due to their increased risk for certain chronic diseases.

## Introduction

Severe acute respiratory syndrome virus 2 (SARS-CoV-2 or COVID-19) is a respiratory tract infection which presents mild symptoms for most. For about 14% of those infected, however, the disease is severe and may require breathing support and time spent in an intensive care unit.^1^ A SARS-CoV-2 outbreak became pandemic on March 11, 2020.^2^ As of April 28, 2021, there were 3,19,24,610 confirmed cases and 569,771 confirmed deaths due to COVID-19 in the United States.^3,4^ The COVID-19 case fatality rate (i.e., deaths among persons with the virus) is 6.48% among military veterans^5^ and 3.07% in the general public in the United States.^6^ The COVID-19 pandemic has placed significant stress on hospitals and clinics,^7^ as well as created the need to better prioritize and implement prevention messaging to the public.^8^

According to the Centers for Disease Control and Prevention (CDC), there are several risk factors for severe COVID-19, including the following underlying conditions: chronic lung disease, asthma, serious heart conditions, obesity, diabetes, and kidney disease.^9^ Additionally, 8002 out of 37,308 deaths due to COVID-19 occur among individuals aged > 64 years.^10^ Studies have shown that persons who have served in the United States military are more likely to report one of these underlying conditions than individuals without military experience.^11–14^ Military veterans are more likely to exhibit one of these underlying conditions because combat experience causes severe mental distress, such as post-traumatic stress disorder (PTSD) and depression, leading to certain unhealthy behaviors that might explain increased prevalence of chronic disease.^11,12,14^ For example, in a study by Albright et al., while 25% of veterans who did not smoke cigarettes felt depressed, 36% of veterans who did smoke cigarettes felt depressed.^15^ Veterans may also be at risk for certain chronic lung diseases due to their exposure to burn pits during military service.^16^

There is a need to better understand where veteran and nonveteran risk factors for severe COVID-19 are highly prevalent, as such an investigation could lead to more targeted COVID-19 prevention messaging efforts tailored for veterans. Although the United States Department of Veterans Affairs has initiated COVID-19 prevention messaging efforts and expanded the use of telemedicine,^17^ their existing efforts could be complemented by geographically targeted prevention messaging guided by an understanding of the prevalence of chronic disease risk factors for severe COVID-19.

In this paper, we use a nationally representative sample of Americans to estimate the prevalence of six risk factors for severe COVID-19 (i.e., chronic obstructive pulmonary disorder [COPD], asthma, diabetes, obesity, cardiovascular disease, and kidney disease) in veterans and nonveterans at the national level, in 155 metropolitan/micropolitan statistical areas, and in Veteran Integrated Service Networks (i.e., for veterans only) in the United States. Our research questions are thus: (1) do veterans and nonveterans have different prevalence rates of the previously described chronic diseases; and (2) does the distribution of chronic diseases among veterans vary by geographic location?

## Methods

### Data collection and sample

**Figure 1.**
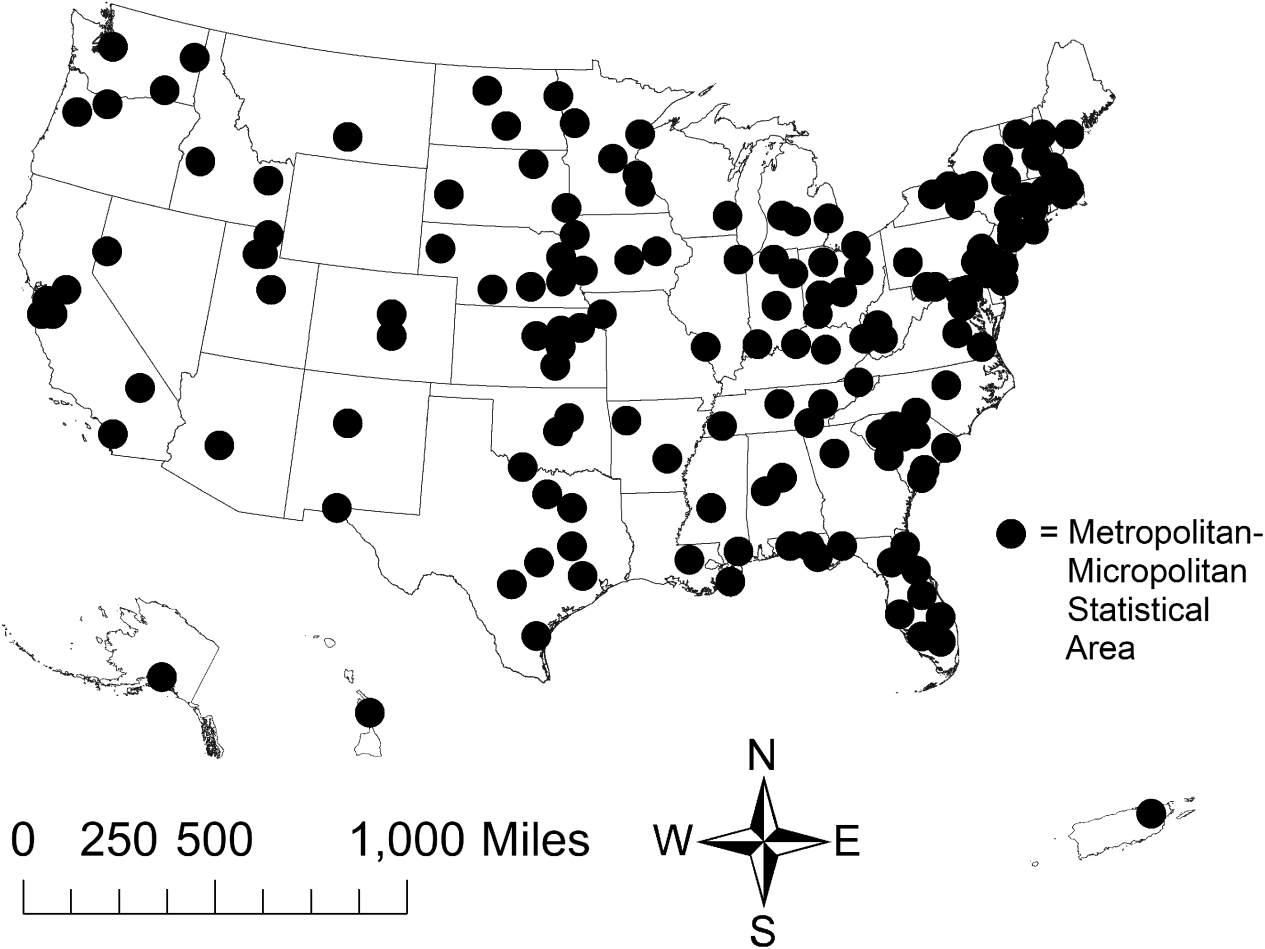
Metropolitan-micropolitan statistical areas (MMSAs) obtained from the Behavioral Risk Factor Surveillance System (BRFSS) between 2015–2017.

We obtained data from the 2015–2017 Selected Metropolitan/Micropolitan Area Risk Trends (SMART) Behavioral Risk Factor Surveillance System (BRFSS) datasets, which provides information on individual-level health behaviors (e.g., alcohol use, cigarette use, physical activity, fruit and vegetable consumption, and seat belt use) and chronic diseases (e.g., COPD, asthma, diabetes, obesity, cardiovascular disease, and kidney disease) for participants in metropolitan or micropolitan areas (MMSAs) with BRFSS samples ≥500.^18^ Participants of the BRFSS are non-institutionalized (i.e., not active duty military living on a military base, or not living in a correctional facility) adults aged > 17 years. A list of areas included in the dataset is provided in Appendix A. After removal of missing data on the measures described below, the final sample included 6,36,527 individuals distributed across 155 MMSAs (Figure 1). The following question was used to determine veteran status: “have you ever served on active duty in the United States Armed Forces, either in the regular military or in a National Guard or military reserve unit?” This question yielded 83,859 veterans and 552,668 nonveterans.

### Measures

We obtained key demographic characteristics about each participant, including age, sex, and race. We selected 6 outcome variables from the SMART BRFSS dataset associated with risk for severe COVID-19, including greater likelihood of death.^9^ Outcome variables in the present study were self-reports of COPD, asthma, diabetes, obesity, cardiovascular disease, and kidney disease. COPD was measured with the following question: “has a doctor, nurse, or other health professional ever told you have chronic obstructive pulmonary disease, COPD, emphysema, or chronic bronchitis (yes or no)?” Asthma was measured with the following question: “has a doctor, nurse, or other health professional ever told you had asthma (yes or no)?” Diabetes was measured with the following question: “has a doctor, nurse, or other health professional ever told you have diabetes (yes or no)?” For purposes of this study, we did not consider gestational diabetes as diagnosed diabetes.

Obesity was determined via calculations of body mass index (BMI) based on self-reported body weight and height. Participants who exhibited a BMI ≥30 were considered obese. Cardiovascular disease (CVD) was determined based on responses to two questions: (1) “has a doctor, nurse, or other health professional ever told you had angina or coronary heart disease”; (2) “has a doctor, nurse, or other health professional ever told you had a heart attack, also called a myocardial infarction?” Participants who answered in the affirmative to either of these questions were considered to have CVD. Lastly, kidney disease was determined using this question: “has a doctor, nurse, or other health professional ever told you have kidney disease (do not include kidney stones, bladder infection, or incontinence)?” Responses to this question included yes or no. Geographic information – that is, the name of the MMSA of residence and it’s MMSA type (i.e., Metropolitan Division, Metropolitan Statistical Area, or Micropolitan Statistical Area) – was also available for each participant.

### Data analysis

Direct survey-weighted prevalence rates for veterans and nonveterans, separately, were developed at the national level, for each MMSA with at least 50 veterans or 50 nonveterans, and in VISNs (for veterans only).^19^ We also generated standard errors for each veteran/nonveteran specific prevalence estimate. Our screening of MMSAs revealed that 1 of 155 MMSAs did not meet the aforementioned inclusion criteria. All results for this MMSA (i.e., Lexington-Fayette, KY) were suppressed. We provide all veteran/nonveteran specific MMSA prevalence rates in Appendix A. We present 3 MMSAs with the highest prevalence rates of each risk factor for COVID-19 for veterans and nonveterans in the results section, as well as compare veteran and nonveteran prevalence rates by metro- and micro-politan statistical areas. We used chi-square analysis or multivariable logistic regression in order to determine differences in our outcomes by veteran status or geography.

## Results

Demographic and health characteristics of the study sample, stratified by veteran status, are given in Table 1. The national unadjusted prevalence – given as a percentage and standard error – of six risk factors for severe COVID-19 in veterans versus nonveterans, respectively, were as follows: COPD, 8.73% ± 0.20 vs. 5.32% ± 0.06 (p < 0.001); asthma, 9.56% ± 0.21 vs. 14.08% ± 0.09 (p < 0.001); diabetes, 16.04% ± 0.26 vs. 9.42% ± 0.08 (p < 0.001); CVD, 14.02% ± 0.23 vs. 5.07% ± 0.06 (p < 0.001); kidney disease, 4.32% ± 0.15 vs. 2.55% ± 0.04 (p < 0.001); and obesity, 29.86% ± 0.33 vs. 27.77% ± 0.12 (p < 0.001).

**Table 1.** Demographic and health characteristics of the study sample by veteran status, SMART BRFSS 2015–2017.

	Veteran(n = 83,859)		Nonveteran(n = 5,52,668)		
Variable	n	%	n	%	p^a^
Age (Years)					
18 to 24	1,441	4.43	36,554	13.36	
25 to 34	4,284	11.01	63,696	18.46	
35 to 44	5,349	10.84	71,260	17.54	
45 to 54	9,828	15.02	93,728	17.72	
55 to 64	14,164	16.37	120,924	16.53	
≥65	48,793	42.33	166,506	16.39	<0.001
Sex					
Male	76,057	90.06	211,666	45.55	
Female	7,779	9.92	340,901	54.43	
Refused	23	0.02	101	0.02	<0.001
Race					
White	70,811	77.11	442,177	69.57	
Black	8,110	14.61	59,858	14.46	
American Indian	1,209	1.43	7,397	1.58	
Asian	608	2.13	14,973	7.01	
Native Hawaiian	228	0.38	1,908	0.40	
Other race	1,120	1.89	13,173	3.45	
No preferred race	244	0.28	1,647	0.32	
Multiracial	2	<0.01	6	<0.01	
Don’t know	300	0.52	4,732	1.45	
Refused	1,227	1.65	6,797	1.74	<0.001
MMSA type					
Metropolitan division	13,801	21.13	110,988	26.57	
Metroplitan statistical area	68,196	78.55	430,181	73.20	
Micropolitan statistical Area	1,862	0.32	11,499	0.24	<0.001
Chonic disease outcomes					
COPD	8,480	8.73	38,876	5.32	<0.001
Asthma	7,760	9.56	78,106	14.08	<0.001
Diabetes	15,566	16.04	64,220	9.42	<0.001
CVD	14,509	14.02	38,742	5.07	<0.001
Kidney disease	4,184	4.32	18,454	2.55	<0.001
Obesity	24,870	29.86	159,334	27.77	<0.001

^a^p-Value for Pearson chi-square statistic.

After adjusting for confounding variables – including age, race, sex, and geographic location – results of multivariable logistic regression models (Table 2), in which the six risk factors for severe COVID-19 served as dependent variables in separate models, showed that veterans were at greater risk for only COPD (aOR = 1.36, p < 0.001), diabetes (aOR = 1.05, p = 0.4), CVD (aOR = 1.37, p < 0.001), and kidney disease (p < 0.001). Veterans were less likely than nonveterans to report asthma after covariate adjustment (aOR = 0.79, p < 0.001).

**Table 2.** Mulivariable logistic regression results comparing likelihood of risk factors for severe COVID-19 in veterans and nonveterans at the national-level (n = 6,36,519).

Variable	COPD aOR (SE)	AsthmaaOR (SE)	DiabetesaOR (SE)	CVDaOR (SE)	Kidney diseaseaOR (SE)	ObesityaOR (SE)
Veteran						
No	(Ref)	(Ref)	(Ref)	(Ref)	(Ref)	(Ref)
Yes	1.36 (0.04)**	0.79 (0.02)**	1.05 (0.03)*	1.37 (0.04)**	1.19 (0.05)**	1.01 (0.02)
MMSA type						
Metropolitan division	(Ref)	(Ref)	(Ref)	(Ref)	(Ref)	(Ref)
Metroplitan statistical area	1.20 (0.03)**	0.97 (0.02)	1.19 (0.02)**	1.13 (0.03)**	1.15 (0.04)**	1.19 (0.02)**
Micropolitan statistical area	1.28 (0.07)**	1.05 (0.05)	1.08 (0.05)	1.10 (0.05)*	0.99 (0.09)	1.30 (0.04)**
Intercept	0.01 (0.01)**	0.20 (0.01)**	0.01 (0.01)**	0.01 (0.01)**	0.01 (0.01)**	0.15 (0.01)**

Note: All models are adjusted for age, sex, and race/ethnicity (i.e., except for races for which too few participants were identified).

*p < 0.05; **p < 0.001.

**Figure 2.**
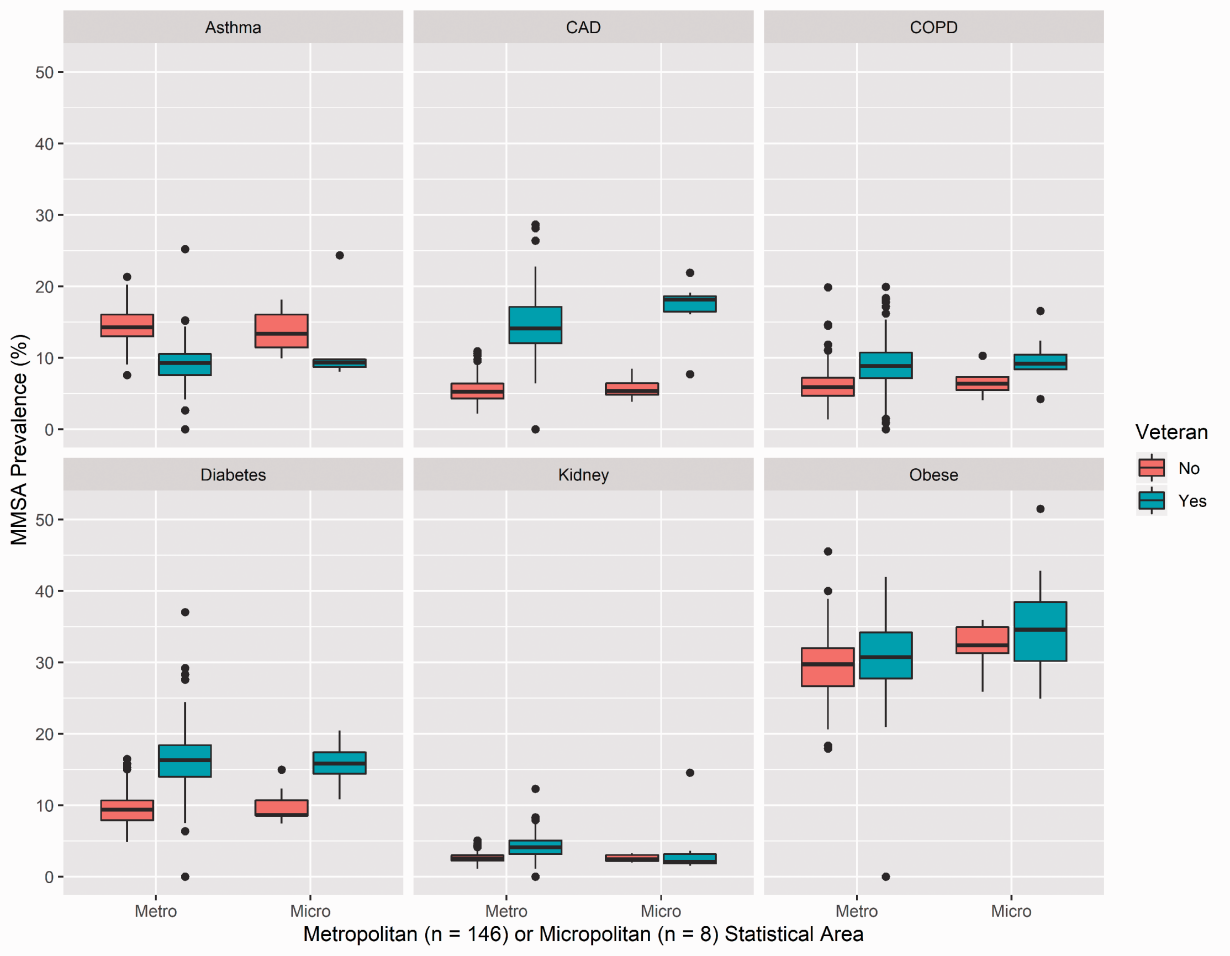
COVID-19 risk factor prevalence rates by MMSA type and veteran status.

Results pertaining to veteran/nonveteran prevalence rates of risk factors for severe COVID-19 in MMSAs are shown in Table 3. Specifically, in Table 3, we present the 3 MMSAs with the highest prevalence of risk factors for severe COVID-19 in veterans and nonveterans, separately. As shown in Table 3, results for nonveterans indicated that, in particular, Beckley, WV, was among the top 3 MMSAs with high prevalence of all risk factors for COVID-19 except for kidney disease. Regarding veterans, Berlin, NH, appeared in the top 3 MMSAs for high prevalence of several COVID-19 risk factors. Furthermore, several MMSAs had veteran prevalence rates more than double the rate of the highest nonveteran rate (i.e., San Antonio-New Braunfels, TX, for diabetes, and Beckley, WV, for CVD). Figure 2 shows that veteran and nonveteran prevalence rates were higher in micropolitan rather than metropolitan areas, except for asthma.

**Table 3.** Metropolitan and micropolitan statistical areas (MMSA) with the highest veteran and nonveteran prevalence rates of risk factors for severe COVID-19, United States, 2015–2017.

	Veteran		Nonveteran	
Variable	MMSA	% ± SE	MMSA	% ± SE
COPD				
1st Highest prevalence	Chattanooga, TN-GA	19.91 ± 6.48	Beckley, WV	19.86 ± 2.27
2nd Highest prevalence	Akron, OH	18.37 ± 7.41	Huntington-Ashland, WV-KY-OH	14.64 ± 0.76
3rd Highest prevalence	Kingsport-Bristol-Bristol, TN-VA	18.21 ± 3.52	Kingsport-Bristol-Bristol, TN-VA	14.50 ± 1.41
Asthma				
1st Highest prevalence	Binghamton, NY	25.22 ± 5.50	Beckley, WV	21.31 ± 2.55
2nd Highest prevalence	Berlin, NH-VT	24.36 ± 9.06	Salem, OR	20.21 ± 2.44
3rd Highest prevalence	Boston, MA	15.26 ± 2.43	Hagerstown-Martinsburg, MD-WV	19.26 ± 1.68
Diabetes				
1st Highest prevalence	San Antonio-New Braunfels, TX	37.04 ± 9.00	Beckley, WV	16.48 ± 1.94
2nd Highest prevalence	Rockingham County-Strafford County, NH	29.20 ± 5.71	Corpus Christi, TX	15.78 ± 1.85
3rd Highest prevalence	Charlotte-Concord-Gastonia, NC-SC	28.31 ± 6.36	Charleston, WV	15.75 ± 0.90
CVD				
1st Highest prevalence	Beckley, WV	28.66 ± 6.15	Wichita Falls, TX	10.91 ± 2.04
2nd Highest prevalence	Idaho Falls, ID	28.13 ± 6.55	Huntington-Ashland, WV-KY-OH	10.88 ± 0.67
3rd Highest prevalence	Cumberland, MD-WV	26.39 ± 6.16	Beckley, WV	10.62 ± 1.59
Kidney disease				
1st Highest prevalence	Berlin, NH-VT	14.54 ± 9.00	Crestview-Fort Walton Beach-Destin, FL	5.05 ± 0.87
2nd Highest prevalence	South Bend-Mishawaka, IN-MI	12.28 ± 4.82	Charleston, WV	4.58 ± 0.48
3rd Highest prevalence	Kingsport-Bristol-Bristol, TN-VA	8.30 ± 2.73	Florence, SC	4.39 ± 0.83
Obesity				
1st Highest prevalence	Lexington-Fayette, KY	Suppressed	Beckley, WV	45.53 ± 2.83
2nd Highest prevalence	Berlin, NH-VT	51.48 ± 8.16	Corpus Christi, TX	40.00 ± 2.96
3rd Highest prevalence	Norfolk, NE	42.80 ± 4.61	Charleston, WV	38.90 ± 1.30

Veteran-specific prevalence rates of the six risk factors for severe COVID-19 by VISN are shown in Table 4. Results for COPD prevalence showed that rates varied by VISN (Χ^2^ = 6.15, p < 0.001), with VISN 9 (i.e., Kentucky, Tennessee) exhibiting the highest rate (12.25%). Results for asthma prevalence showed that rates did not vary by VISN (Χ^2^ = 1.36, p = 0.18). Results for diabetes prevalence showed that rates varied by VISN (Χ^2^ = 2.12, p = 0.02), with VISN 9 exhibiting the highest rate (18.96%). Results for CVD prevalence showed that rates varied by VISN (Χ^2^ = 5.34, p < 0.001), with VISN 1 (i.e., Maine, Vermont, New Hampshire, Massachussettes, Connecticut, Rhode Island) exhibiting the highest rate (17.94%). Prevalence rates for kidney disease did not vary by VISN (Χ^2^ = 1.48, p = 0.13). Finally, results for obesity showed that rates varied by VISN (Χ^2^ = 4.60, p < 0.001), with VISN 9 exhibiting the highest rate (34.99%).

**Table 4.** Risk factors for severe COVID-19 among military veterans by Veteran Integrated Service Network (VISN) in the United States (n = 83,859).

VISN^a^	COPD n (%)	Asthma n (%)	Diabetes n (%)	CVD n (%)	Kidney disease n (%)	Obesity n (%)
1	946 (9.28)	920 (11.28)	1,585 (16.07)	1,663 (17.94)	400 (4.33)	2,463 (28.48)
2	488 (9.20)	413 (9.64)	882 (17.76)	845 (16.26)	231 (4.70)	1,232 (27.02)
4	271 (10.93)	219 (9.33)	456 (17.47)	411 (15.48)	104 (4.69)	715 (31.78)
5	682 (6.24)	696 (8.21)	1,441 (14.34)	1,259 (11.01)	391 (3.28)	2,297 (27.55)
6	237 (7.27)	231 (8.55)	500 (13.72)	356 (10.13)	123 (3.57)	838 (30.68)
7	514 (8.57)	486 (10.45)	991 (16.49)	853 (13.05)	272 (4.37)	1,520 (31.04)
8	624 (8.80)	442 (8.46)	1,095 (14.82)	1,058 (15.13)	292 (4.03)	1,650 (27.96)
9	358 (12.25)	267 (10.47)	565 (18.96)	534 (16.89)	161 (4.92)	824 (34.99)
10	528 (10.71)	431 (10.37)	956 (17.77)	918 (16.52)	277 (4.98)	1,499 (32.99)
12	208 (10.98)	162 (10.45)	341 (18.07)	318 (16.53)	105 (5.69)	507 (32.16)
15	650 (11.01)	548 (11.30)	1,154 (16.87)	1,035 (14.89)	303 (4.55)	1,924 (34.40)
16	328 (11.45)	240 (9.43)	564 (17.26)	518 (12.73)	160 (4.52)	862 (32.89)
17	175 (5.24)	196 (9.27)	435 (15.75)	378 (11.78)	103 (3.00)	658 (31.11)
19	604 (8.61)	638 (9.69)	1,115 (14.12)	1,034 (12.34)	319 (3.68)	1,871 (27.93)
20	431 (8.00)	493 (10.57)	837 (14.91)	773 (13.07)	231 (4.08)	1,391 (30.87)
21	111 (5.25)	160 (10.46)	223 (14.02)	192 (12.45)	71 (4.62)	322 (24.37)
22	438 (7.92)	465 (9.03)	807 (15.90)	767 (12.99)	249 (5.11)	1,211 (25.98)
23	887 (7.61)	753 (7.34)	1,619 (14.06)	1,597 (13.52)	392 (3.76)	3,086 (32.19)
Χ^2^ (df)	6.15 (9.57, 8.0e + 05)***	1.36 (10.90, 9.1e + 05)	2.12 (9.89, 8.3e + 05)*	5.34 (10.95, 9.2e + 05)***	1.48 (10.73, 9.0e + 05)	4.60 (11.28, 9.5e + 05)***

*p < 0.05; **p < 0.01; ***p < 0.001.

^a^States (abbreviated) included in each VISN: (1) ME, VT, NH, MA, CT, RI; (2) NY, NJ; (4) PA, DE, NJ, OH, (5) MD, WV, VA, KY, DC; (6) NC, VA; (7) SC, GA, AL; (8) FL; (9) KY, TN; (10) MI, IN, OH, KY; (12) WI, IL, IN, MI; (15) KS, MO, IL, IN, KY; (16) AR, MS, LA; (17) TX; (19) MT, WY, CO, UT, OK, NV; (20) WA, OR, ID, AK, MT; (21) CA, NV, HI; (22) AZ, NM, CA, NV; (23) ND, MN, SD, NE, IA, WI, IL.

## Discussion

Our results suggest that, in the national aggregate, veterans may exhibit higher prevalence rates of risk factors for severe COVID-19 than nonveterans, except for asthma and obesity. However, we demonstrated that there is significant variation in the prevalence of risk factors for severe COVID-19 among veterans and nonveterans by geographic location. While the MMSAs with the top 3 highest prevalence rates for various risk factors for severe COVID-19 in veterans and nonveterans were similar for some risk factors (e.g., COPD in Kingsport-Bristol-Bristol, TN-VA, and CVD in Beckley, WV), many risk factors had completely different MMSA mixes by veteran status (e.g., there were no shared top 3 MMSAs for asthma between veterans and nonveterans). Furthermore, among veterans only, we showed that prevalence rates for COPD, diabetes, CVD, and obesity varied by VISN, with VISN’s 1 (CVD) and 9 (COPD, diabetes, and obesity) being at greatest risk.

Our study of veterans coincides with other studies which have shown (a) that veterans have higher rates of chronic disease than nonveterans^20^ and (b) that the distribution of chronic disease varies by geographic location.^21,22^ Specifically, as we found in our study, areas that are less urbanized are more likely to have higher prevalence rates of chronic disease than areas that are highly urbanized. However, our study advances the literature in that we examine veteran-nonveteran differences in chronic disease prevalence by geographic location, instead of examining these differences only in the national aggregate or by only looking at veterans in isolation. This understanding is critically important in the context of the COVID-19 pandemic, as the chronic diseases investigated in this paper are risk factors for severe COVID-19 and our analyses could result in the geographic targeting of prevention messaging for veterans.

Public health efforts aimed at veterans, especially as it concerns the prevention of severe COVID-19, should consider geography in the programmatic formula. For example, the veteran prevalence rate of diabetes in the MMSA with the highest rate was more than double that of the prevalence rate of diabetes in the MMSA with the highest nonveteran rate. Therefore, public health messaging efforts within the Department of Veterans Affairs may be directed toward geographic areas at greatest risk for the development of severe COVID-19 (i.e., areas with highly prevalent chronic diseases among veterans).

Some limitations accompanied the analysis of data in this study. First, the number of MMSAs included in this study was not exhaustive. For example, we were not able to obtain MMSAs from Wyoming. Second, all data in this study was based on self-report. Because we were unable to obtain data from medical records to confirm particular diagnoses, the prevalence rates in our study may be underestimated, particularly due to social desirability bias or recall bias. Third, one MMSA had a veteran sample < 50. As such, we were unable to display estimates from that particular MMSA, largely due to the instability of the estimate. Fourth, we do not account for and provide MMSA-level estimates of many other factors related to severe COVID-19 outcomes, such as social inequality.

In conclusion, the present study provides a searchable database of MMSA-level and VISN-level prevalence rates for various risk factors for severe COVID-19 by veteran status. Because veterans and nonveteran often seek care from different healthcare sources, there is a need to understand where public health messaging within each healthcare system should be directed during the COVID-19 pandemic. The MMSA-specific results of this study (i.e., proportions of all chronic disease outcomes by veteran status are given in Appendix A) could be used by the Department of Veterans Affairs (VA) and other private/public healthcare providers in order to plan for COVID-19 prevention. For example, the VA could develop a social media message such as, “If you have diabetes, you may be at risk for a severe case of COVID-19; as such, getting the COVID-19 vaccine could be particularly important for you in terms of preventing a sever case of COVID-19,” for an MMSA or VISN that has a relatively high rate of diabetes. Future studies should examine veteran-nonveteran differences in COVID-19 illness severity by geographic region.

## Supplemental Material

sj-xlsx-1-chi-10.1177_17423953211028280 - Supplemental material for Examining the geographic distribution of six chronic disease risk factors for severe COVID-19: Veteran–nonveteran differences Click here for additional data file. Supplemental material, sj-xlsx-1-chi-10.1177_17423953211028280 for Examining the geographic distribution of six chronic disease risk factors for severe COVID-19: Veteran–nonveteran differences by Michelle McDaniel and Justin T McDaniel in Chronic Illness
